# ELK3 Mediated by ZEB1 Facilitates the Growth and Metastasis of Pancreatic Carcinoma by Activating the Wnt/β-Catenin Pathway

**DOI:** 10.3389/fcell.2021.700192

**Published:** 2021-08-02

**Authors:** Qiuyan Zhao, Yingchun Ren, Haoran Xie, Lanting Yu, Jiawei Lu, Weiliang Jiang, Wenqin Xiao, Zhonglin Zhu, Rong Wan, Baiwen Li

**Affiliations:** ^1^Department of Gastroenterology, Shanghai General Hospital, Shanghai Jiao Tong University School of Medicine, Shanghai, China; ^2^Shanghai Key Laboratory of Pancreatic Diseases, Shanghai General Hospital, Shanghai Jiao Tong University School of Medicine, Shanghai, China; ^3^Department of Gastrointestinal Surgery, Henan Provincial People’s Hospital, Zhengzhou, China

**Keywords:** pancreatic ductal adenocarcinoma, ELK3, EMT, Wnt/β-catenin, ZEB1

## Abstract

Rapid progression and metastasis are the major causes of death in patients with pancreatic ductal adenocarcinoma (PDAC). ELK3, a member of the ternary complex factor (TCF), has been associated with the initiation and progression of various cancers. However, the role of ELK3 in PDAC is not yet fully understood. Online databases and immunohistochemistry were used to analyze the ELK3 levels in PDAC tissues. The function of ELK3 was confirmed by a series of *in vivo* and *in vitro* studies. Western blotting and immunofluorescence were used to detect the molecular mechanisms of PDAC. ChIP-qPCR was used to study the mechanism responsible for the elevation of ELK3 expression in PDAC. The ELK3 levels were higher in PDAC tissues than in adjacent normal tissues. Functionally, we demonstrated that ELK3 acted as an oncogene to promote PDAC tumorigenesis and metastasis. Further study suggested that ELK3 promoted PDAC cell migration and invasion by activating the Wnt/β-catenin pathway, and proved that ZEB1 could directly bind to the promoter of ELK3 to increase its transcription. Finally, both were associated with the patients’ clinicopathological features and worse overall survival. Conclusively, our findings enrich the role of ELK3 in PDAC, and provide potential avenues for exploring more effective biomarkers and therapeutic strategies for the treatment of PDAC.

## Introduction

Pancreatic ductal adenocarcinoma (PDAC) remains an intractable disease with a 5-year survival rate of 9%, which is the lowest among the different types of cancer (Siegel et al., 2020). Due to lack of effective screening tools for early detection, most patients are diagnosed with metastatic or advanced tumor, while the vast majority of patients relapse within 1 year, even in the early stage of disease after surgical resection (Kamisawa et al., 2016). Rapid progression and distant metastasis are the leading causes of poor prognosis in PDAC patients (Ryan et al., 2014; Klein, 2019). Therefore, it is paramount to identify the underlying molecular pathophysiology and effective therapeutic targets.

ELK3 is a member of the ternary complex factor (TCF), a subfamily of ETS domain transcription factors (Maira et al., 1996). The function of ELK3 is capable of forming a ternary complex with serum response factor (SRF) to regulate gene expression (Nozaki et al., 1996; Li et al., 2000). Several studies have demonstrated that ELK3 is associated with the initiation and progression of various cancers. For instance, ELK3 suppression results in extensive changes in the expression profiles of breast cancer, thus decreases cell migration and metastasis during tumor progression (Kong et al., 2016). In squamous cell carcinoma (SCC), ELK3 knockdown severely impairs tumor growth and prohibits progression from benign papillomas to SCC (Yang et al., 2015). In addition, ELK3 has also been proved to be associated with chemoresistance (Wang et al., 2019), angiogenesis and wound closure (Robertson et al., 2014). Although increasing evidence has demonstrated the biological importance of ELK3, its role in PDAC and the potential molecular mechanisms have not been fully elucidated.

Epithelial-mesenchymal transition (EMT) is an essential biological process associated with tumor metastasis, wherein epithelial cells alter their shape, modify the adhesion molecules, and acquire migratory and invasive behaviors (Reymond et al., 2013; David et al., 2016; Nieto et al., 2016; Lambert et al., 2017). The EMT process is driven by multiple transcription factors, notably Twist, Zeb1, bHLH, Snail and Slug, which orchestrate and coordinate to repress the epithelial marker E-cadherin and activate the expression of mesenchymal markers such as N-cadherin and Vimentin (Thiery, 2002; De Craene and Berx, 2013). The reprogramming of expression of these genes are initiated and controlled by multiple signaling pathways, and among these, Wnt/β-catenin plays a critical role in the induction of EMT (Lamouille et al., 2014; Dongre and Weinberg, 2019). Once the canonical Wnt pathway is activated, β-catenin translocates to the nucleus, where it complexes with T cell factor and lymphoid enhancer binding factor (TCF-LEF) to activate the transcription of genes that favor EMT (Nelson and Nusse, 2004; Townsley et al., 2004; Hoffmans et al., 2005). Studies have shown that approximately 80% of colorectal carcinoma has nuclear accumulation of β-catenin, which is associated with poor prognosis (Martensson et al., 2007; Li et al., 2017). Consistently, the hyperactivation of the Wnt/β-catenin signaling pathway increases susceptibility to hepatocellular carcinoma development (Tao et al., 2016). However, the role of the Wnt/β-catenin pathway in PDAC is less distinct and somewhat controversial (White et al., 2012). Hence, it is necessary to explore the molecular mechanisms of Wnt/β-catenin signaling in PDAC.

In the present study, we first identified the upregulation of ELK3 in pancreatic cancer tissues. We demonstrated ELK3 is an oncogenic gene that accelerates PDAC cells proliferation and invasion through activating β-catenin signaling pathway. Furthermore, we proved that ELK3 is regulated by ZEB1, which binds to the promoter of ELK3 and activates its expression. In clinic, ZEB1 is also upregulated in PDAC, and both are related to tumor progression and poor survival in PDAC patients.

## Materials and Methods

### Cell Lines

The human pancreatic cancer cell lines PANC-1 and MIA PaCa-2 were obtained from the Type Culture Collection of the Chinese Academy of Science (Shanghai, China). Both were cultured in DMEM (Gibco) supplemented with 10% fetal bovine serum (FBS; Gibco) and 1% penicillin-streptomycin at 37°C with 5% CO2.

### Tissue Microarray (TMA)

The clinical characteristics of ELK3 and ZEB1 expression in PDAC patients were analyzed using TMAs containing 70 pairs of pancreatic cancer samples, purchased from Shanghai Outdo Biotech (Shanghai, China). TMAs contain patients’ complete clinicopathological information and follow-up data. The application of TMAs complied with relevant regulations, and present study was approved by the Ethics Committee of Shanghai General Hospital.

### Immunohistochemistry (IHC)

After deparaffinization and dehydration, the TMAs were boiled in sodium citrate solution (0.01 M, pH 6.0) for 15 min. Next, 3% hydrogen peroxide was used to block endogenous peroxidase activity. Next, the TMAs were incubated with ELK3 antibody (1:200, Sigma HPA001600) and ZEB1 antibody (1:200, Sigma HPA027524), respectively, at 4°C overnight. The following day, the TMAs were incubated with secondary antibodies for 1 h at room temperature. IHC staining scores were based on staining intensity and staining area. The staining intensity was divided into four levels: 0 (negative staining), 1 (weak staining), 2 (moderate staining), and 3 (strong staining). Staining areas were scored as 0 (0–10%), 1 (10–25%), 2 (25–50%), 3 (50–75%), and 4 (75–100%). The final score was calculated by multiplying the above two scores. Total score of ≤ 4 indicated low expression and > 4 indicated high expression. Assessed by three proficient pathologists independently, the score applied for both ELK3 and ZEB1 expression.

### Construction of Stable Knockdown and Overexpressed Cell Lines

Stable knockdown and overexpression of ELK3 were achieved by construction of lentivirus vector (OBiO Biotechnology, Shanghai, China). PANC-1 and MIA PaCa-2 cells were cultured in 6-well plates. When the cells reached 70% confluence, they were infected with appropriate lentiviruses in the presence of 6μg/ml polybrene (Hanbio Biotechnology, Shanghai, China). Infected cells were selected using 4 μg/ml puromycin (Sigma, United States) for 2 weeks. Transfection efficiency was determined by qRT-PCR and western blotting analysis.

### Plasmid Construction, RNAi and Cell Transfection

The overexpression vector and small interfering RNAs (siRNAs) specifically targeting genes were synthesized by RiboBio (Guangzhou, China). Transfection of plasmids or siRNAs in pancreatic cancer cells was performed using Lipofectamine^TM^2000 (Invitrogen, United States 11668-019) following the manufacturer’s instructions. The transfection efficiency was determined by qRT-PCR and western blotting analysis.

### RNA Extraction and Quantitative Real-Time Polymerase Chain Reaction (qRT-PCR)

Total RNA was isolated from cells using RNAiso Plus Reagent (TakaRa) and was reverse-transcribed to cDNA using PrimeScript^TM^ RT reagent kit (TakaRa). Then, SYBR^®^ Premix Ex Taq^TM^ (TakaRa) was used to amplify cDNA. Glyceraldehyde 3-phosphate dehydrogenase (GAPDH) was used as the internal control to normalize data. The relative gene expression of mRNAs was determined by the 2^–ΔΔCt^ method.

### Western Blotting Analysis

Pancreatic cancer cells were lysed in RIPA buffer containing protease and phosphatase inhibitors. BCA Protein Assay Kit (Beyotime Biotechnology, China) was used to measure the concentration of protein. Protein samples were separated by SDS-PAGE at 90 V for 2 h and then transfected into PVDF membranes for 2 h. After blocking in 5% fat-free milk for 1.5 h, the membranes were incubated with primary antibodies at 4°C overnight. Next day, the membranes were incubated with secondary antibodies for 2 h. Finally, the membranes washed using TBST and detected by ECL chemiluminescent reagent (Millipore, United States).

### Cell Wound Healing Assay

Pancreatic cancer cells were cultured in 6-well plates. When the cells grown to full confluence, a scratch wound was made in the center of the well using a 200 μl plastic micropipette tip. Wound healing images were captured at 0 and 24 h after injury. The width of wound healing was quantified and compared with baseline values.

### Cell Migration and Invasion Assays

Cancer cells (2–5 × 10^4^) in serum-free medium were seeded into upper chamber of 24-well plates (Corning, United States) with an 8.0 μm pore size polycarbonate membrane without (migration) or with (invasion) matrigel. The medium in the bottom chamber contained 10% FBS as the chemoattractant. After 24 h, the cells in the upper chamber were removed using a cotton swab. The cells that migrated and invaded to lower chamber were stained with 0.1% crystal violet and counted in five random fields under a microscope.

### Confocal Immunofluorescence Assay

When cells seeded in the confocal plates grown into 50–60% confluence, they were washed with PBS, fixed in 4% paraformaldehyde for 20 min, permeabilized with 0.1% Triton X-100 for 20 min and blocked with 5% goat serum for 1 h at room temperature. The cells were then incubated with primary antibodies at 4°C overnight followed by incubation with fluorophore-conjugated secondary antibody for 1 h. Following washing, the samples were stained with DAPI and imaged using a confocal microscope (Leica, Germany). The primary antibodies were listed as follows: E-cadherin (1:100, Cell Signaling Technology 3195S), N-cadherin (1:100, Cell Signaling Technology 13116S), Vimentin (1:250, Abcam ab92547), and β-catenin (1:250, Abcam ab32572).

### Chromatin Immunoprecipitation (ChIP) Assay

ChIP experiments were performed using 1 × 10^7^ pancreatic cancer cells with the ChIP kit (Cell Signaling Technology) according to the manufacturer’s protocol. Briefly, PDAC cells were cross-linked with 1% formaldehyde. Then the cells were lysed and chromatin was harvested and fragmented using enzymatic digestion. The chromatin was immunoprecipitated using ChIP-grade antibody against ZEB1 (Sigma, United States). After immunoprecipitation, the protein-DNA cross-links were reversed and the DNA was purified, followed by qRT-PCR. The enrichment was calculated using the following formula: percentage = 2% × 2^(C[T]Input Sample^^–^^C[T]^ ^IP Sample)^. The qRT-PCR products were used for DNA electrophoresis and visualized by ethidium bromide staining.

### Luciferase Reporter Assay

The luciferase reporter plasmids (pGL3-Luc containing the ELK3 promoter, pGL3-Luc containing intact binding site#2 in the ELK3 promoter and pGL3-Luc containing mutant binding site#2 in the ELK3 promoter) were synthesized by GenePharma (Shanghai, China). PDAC cells were co-transfected with approximately 80 ng of luciferase reporter plasmids and 100 ng of si-ZEB1 or ZEB1 overexpression vectors using Lipofectamine^TM^ 2000 (Invitrogen, United States). After incubation for 36 h, luciferase activities were detected using the Dual Luciferase Reporter Assay System (Promega, United States) and calculated with the ratio of firefly luciferase/Renilla luciferase activity.

### Animal Experiments

The animal experiments in this study were approved by the Animal Care Committee of Shanghai General Hospital. To study primary tumor growth *in vivo*, 4-week-old male BALB/c nude mice were chosen and maintained under specific pathogen-free conditions. The mice were randomly divided into four groups (*n* = 5), and stable cell lines (1.0 × 10^7^) were subcutaneously injected into the armpit of the nude mice. Tumor volumes were estimated in accordance with the formula (length × width^2^)/2 and measured twice a week. After 21 days, the mice were sacrificed and subcutaneous tumors were removed and weighed. For *in vivo* lung metastasis model, the nude mice were randomly divided into four groups (*n* = 5), and 2 × 10^6^ cells were injected through the tail vein. Six weeks later, all of the mice were sacrificed, and the lungs were removed, imaged, paraffin-embedded and stained with hematoxylin and eosin (H&E).

### Statistical Analysis

SPSS 22.0 was conducted for statistical analysis. Significant correlations between ELK3/ZEB1 expression and clinicopathological features of PDAC patients were analyzed by Student’s *t*-test or the Mann-Whitney *U*-test and Pearson *χ*^2^-test. Kaplan-Meier method and log-rank test was used to evaluate survival differences. *P* < 0.05 was considered statistically significant for all tests.

## Results

### ELK3 Is Highly Expressed in PDAC

**FIGURE 1:**
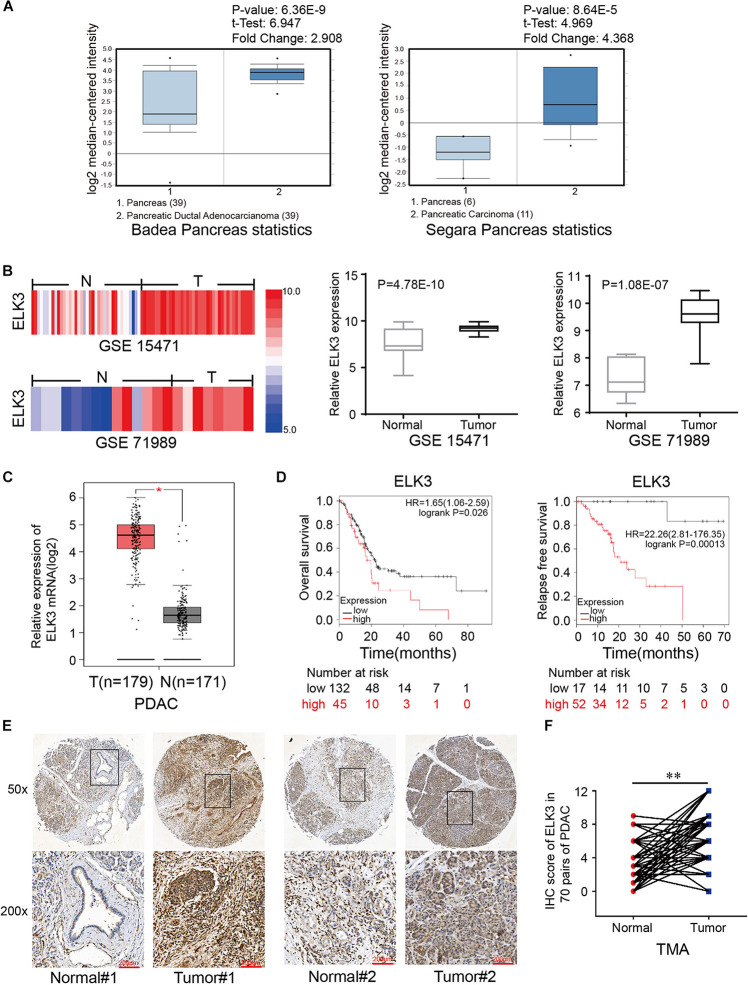
ELK3 is elevated in pancreatic cancer tissues compared with adjacent normal tissues. **(A)** ELK3 expression levels from the Oncomine database. **(B)** ELK3 mRNA levels in GEO datasets GSE15471 (T, *n* = 36; *N*, *n* = 36) and GSE71989 (T, *n* = 14; *N, n* = 8). **(C)** ELK3 mRNA expression in the TCGA cohort (T, *n* = 179; *N, n* = 171). **(D)** Overall survival and relapse free survival of patients with low and high ELK3 levels from the Kaplan-Meier analysis. **(E)** Representative IHC images of ELK3 on the TMA constructed from 70 pancreatic cancer tissues and adjacent normal tissues (scale bar: 200 μm; magnification: top 50× and bottom 200×). **(F)** IHC scores of ELK3 in 70 cases of PDAC tissues with corresponding normal tissues. *P < 0.05, ^∗∗^*P* < 0.01.

To clarify the role of ELK3 in PDAC, we first analyzed the expression of ELK3 using the Oncomine database. The mRNA level of ELK3 was significantly upregulated in pancreatic cancer tissues in two datasets (Badea Pancreas Statistics, *P* = 6.36E-9; Segara Pancreas Statistics, *P* = 8.46E-5) (Figure 1A). Furthermore, the overexpression of ELK3 in PDAC was confirmed by analyzing two NCBI Gene Expression Omnibus (GEO) datasets (GSE15471, *P* = 4.78E-10; GSE71987, *P* = 1.08E-7) (Figure 1B) and The Cancer Genome Atlas (TCGA) dataset (*P* < 0.05) (Figure 1C). The Kaplan-Meier curve analyses revealed that elevated ELK3 level indicated poorer overall survival (OS) and relapse free survival (RFS) (*P* < 0.05) (Figure 1D). To further determine the expression level of ELK3, immunohistochemical (IHC) analysis of the tissue microarray (TMA) containing 70 PDAC tissues and corresponding normal tissues were conducted. As shown in Figures 1E,F, the expression level of ELK3 was higher in PDAC tissues than in adjacent normal tissues. Overall, we conclude that ELK3 is frequently elevated in PDAC, and the role of ELK3 remains to be explored.

### ELK3 Promotes PDAC Cells Proliferation, Migration and Invasion *in vitro*

**FIGURE 2:**
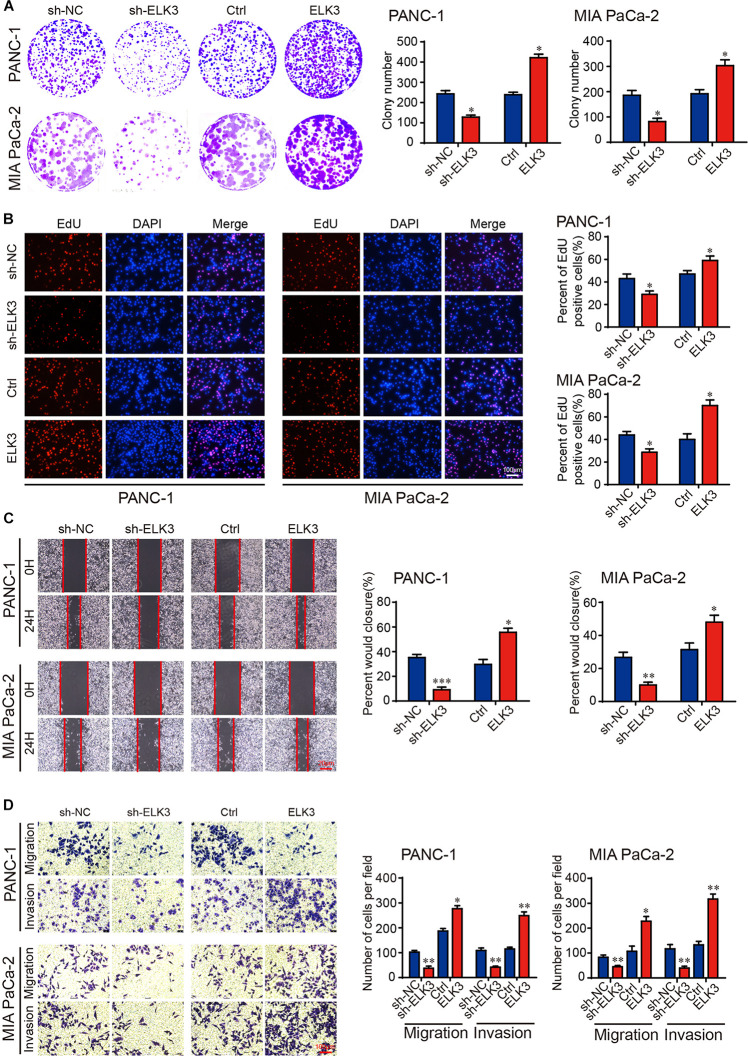
ELK3 promotes proliferation, migration and invasion of PDAC cells *in vitro*. **(A)** Colony formation assay revealing the proliferative ability of indicated PANC-1 and MIA PaCa-2 cells. **(B)** EdU assay confirming the proliferative ability of indicated PANC-1 and MIA PaCa-2 cells (scale bar: 100 μm). **(C)** Representative images of wound healing assays performed with the indicated PANC-1 and MIA PaCa-2 cells (scale bar: 20 μm). **(D)** Representative images of transwell migration and matrigel invasion assays performed with the indicated PANC-1 and MIA PaCa-2 cells (scale bar: 100 μm). Biological triplicate experiments were performed for each group. All data are presented as the mean ± SD. ^∗^*P* < 0.05, ^∗∗^*P* < 0.01, ^∗∗∗^*P* < 0.001.

To investigate the functional roles of ELK3 in PDAC, ELK3 expression was verified by qRT-PCR. The result showed that ELK3 was overexpressed in several pancreatic cancer cells compared with normal pancreatic ductal epithelial cell (HPDE6-c7) (Supplementary Figure 1A). PANC-1 and MIA PaCa-2 cells were selected to knock down and overexpress ELK3, which have been used in our previous experiments (Zhao et al., 2018). We constructed three shRNAs (sh-1, sh-2, sh-3) and a lentiviral overexpression vector targeting ELK3. The qRT-PCR results showed that ELK3 levels were significantly down-regulated or up-regulated in PDAC cells transfected with the indicated shRNAs or overexpression vector, respectively (Supplementary Figure 1B). Among the three shRNAs, sh-1 (sh-ELK3) was selected for further study because it had the highest inhibitory efficiency. In addition, the successful knockdown and overexpression of ELK3 were confirmed at protein levels (Supplementary Figure 1C). Colony formation and EdU assays revealed that knockdown of ELK3 suppressed the proliferation of both PANC-1 and MIA PaCa-2 cells (Figures 2A,B). Conversely, overexpression of ELK3 had the opposite effect on cell proliferation (Figures 2A,B). The wound-healing assay demonstrated that ELK3 depletion inhibited the mobility of PANC-1 cells, while forced expression of ELK3 increased the migration speed of them (Figure 2C). Correspondingly, the effect was confirmed by transwell migration and matrigel invasion assays (Figure 2D). Additionally, similar results were obtained in MIA PaCa-2 cells (Figures 2C,D). In conclusion, our findings indicate that ELK3 may be involved in cell proliferation, migration and invasion, acting as a positive regulator.

### ELK3 Promotes Pancreatic Tumor Growth and Metastasis *in vivo*

**FIGURE 3:**
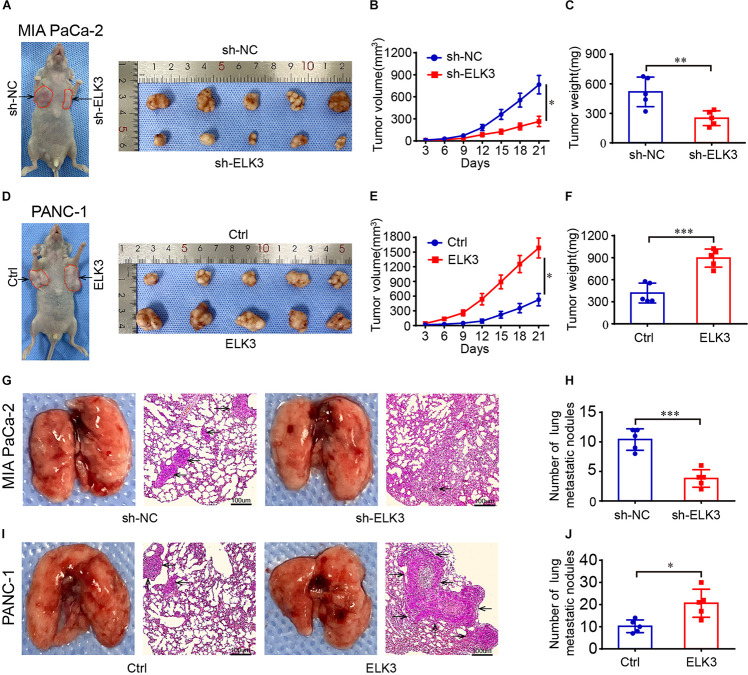
ELK3 promotes tumor growth and metastasis of PDAC cells *in vivo*. **(A)** Representative images of nude mice and xenograft tumors derived from sh-ELK3/MIA PaCa-2 and sh-NC/MIA PaCa-2 cells. **(B)** Tumor growth curves showing the tumor volume, monitored every 3 days in the control and ELK3 knockdown groups. **(C)** The tumors were removed and weighed after 21 days in the control and ELK3 knockdown groups. **(D)** Representative images of nude mice and xenograft tumors derived from Ctrl/PANC-1 and ELK3/PANC-1 cells. **(E)** Tumor growth curves showing the tumor volume as monitored every 3 days in the control and ELK3 overexpressing groups. **(F)** The tumors were removed and weighed after 21 days in the control and ELK3 overexpressing groups. **(G)** Representative photographs of pulmonary metastasis and H&E-stained lung sections in the control and ELK3 knockdown groups (scale bar: 100 μm; magnification: 50×). **(H)** Number of lung metastatic nodules was counted. **(I)** Representative photographs of pulmonary metastasis and H&E-stained lung sections in the control and ELK3 overexpressing groups (scale bar: 100 μm; magnification: 50×). **(J)** Number of lung metastatic nodules was counted. Biological triplicate experiments were performed for each group. All data are presented as the mean ± SD. ^∗^*P* < 0.05, ^∗∗^*P* < 0.01, ^∗∗∗^*P* < 0.001.

To verify the function of ELK3 in pancreatic tumor growth and metastasis *in vivo*, we injected pancreatic cancer cells with stable knockdown or overexpression of ELK3 into the armpit or tail vein of nude mice. The results showed that tumors in the sh-ELK3/MIA PaCa-2 group grew more slowly than those in sh-NC/MIA PaCa-2 group, and this phenomenon was also reflected by tumor volume and final tumor weight (Figures 3A–C). Additionally, the volume and weight of xenograft tumors in ELK3/PANC-1 group were significantly higher than the control tumors of PANC-1 cells (Figures 3D–F). In the lung metastasis model, we discovered that the number of metastatic nodules in mice injected with sh-ELK3/MIA PaCa-2 cells were less than in mice injected with sh-NC/MIA PaCa-2 cells (Figures 3G,H), while a higher number of metastatic nodules was observed in mice injected with ELK3/PANC-1 cells than in those injected with Ctrl/PANC-1 cells (Figures 3I,J). Taken together, these *in vivo* results suggest that ELK3 plays an important role in pancreatic tumor growth and metastasis.

### ELK3 Is Required in TGFβ-Induced EMT

**FIGURE 4:**
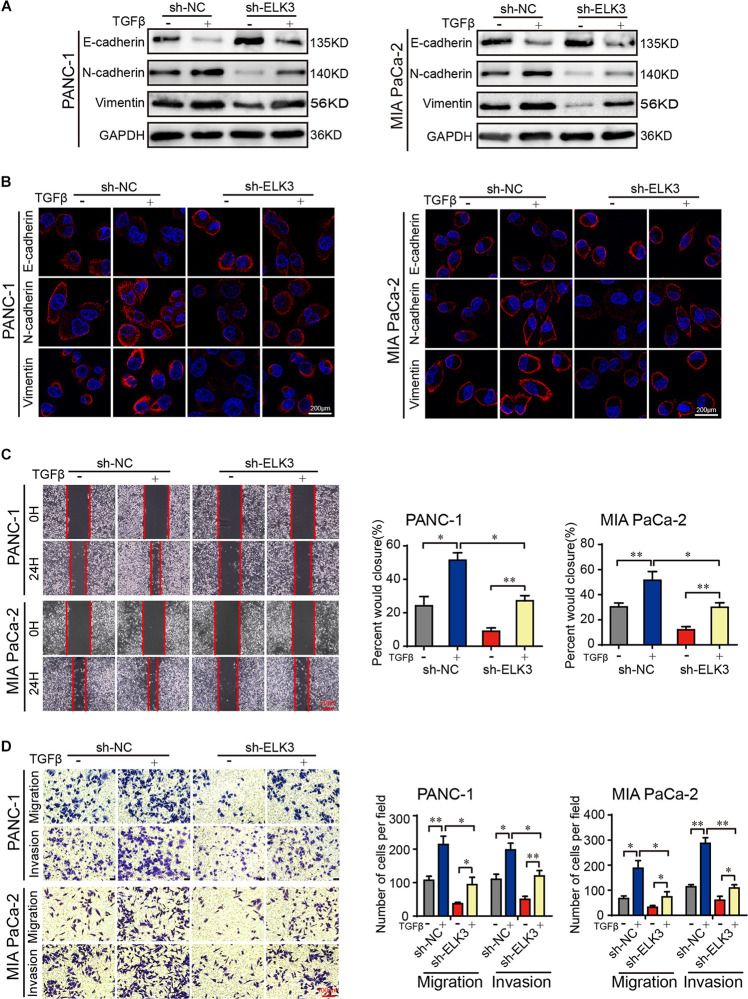
ELK3 is crucial for TGFβ-induced EMT in PDAC. **(A)** Western blot analysis of E-cadherin, N-cadherin and Vimentin expression in the indicated cells. **(B)** Representative confocal immunofluorescence images of E-cadherin, N-cadherin and Vimentin expression in the indicated cells (scale bar: 200 μm). **(C)** Representative images of wound healing in sh-ELK3/PANC-1 and sh-ELK3/MIA PaCa-2 cells treated with or without TGFβ (scale bar: 20 μm). **(D)** Representative images of migration and invasion in sh-ELK3/PANC-1 and sh-ELK3/MIA PaCa-2 cells treated with or without TGFβ (scale bar: 100 μm). Biological triplicate experiments were performed for each group. All data are presented as the mean ± SD. ^∗^*P* < 0.05, ^∗∗^*P* < 0.01.

As we all know, EMT process plays important roles in cancer cell invasion and tumor metastasis (De Craene and Berx, 2013). TGFβ is a potent inducer of EMT, and TGFβ stimulation can irritate changes in cell morphology and biological behavior (Neuzillet et al., 2015; Su et al., 2020). Our results showed that ELK3 was associated with malignant progression of pancreatic cancer. This prompted us to explore the underlying effects of ELK3 on TGFβ-induced EMT process. Western blot and confocal immunofluorescence analysis indicated that TGFβ treatment markedly decreased E-cadherin and increased N-cadherin and Vimentin expression (Figures 4A,B). However, in sh-ELK3/PANC-1 and sh-ELK3/MIA PaCa-2 cells simultaneously treated with TGFβ, these molecular events induced by TGFβ were abolished by ELK3 depletion (Figures 4A,B). Additionally, ELK3 knockdown also effectively quenched the wound healing, cell migration and invasion abilities induced by TGFβ in PANC-1 and MIA PaCa-2 cells (Figures 4C,D). These results demonstrate that ELK3 is crucial for TGFβ-induced EMT in PDAC cells.

### ELK3 Promotes the Progression of Pancreatic Cancer Cells Through the Wnt/β-Catenin Signaling Pathway

**FIGURE 5:**
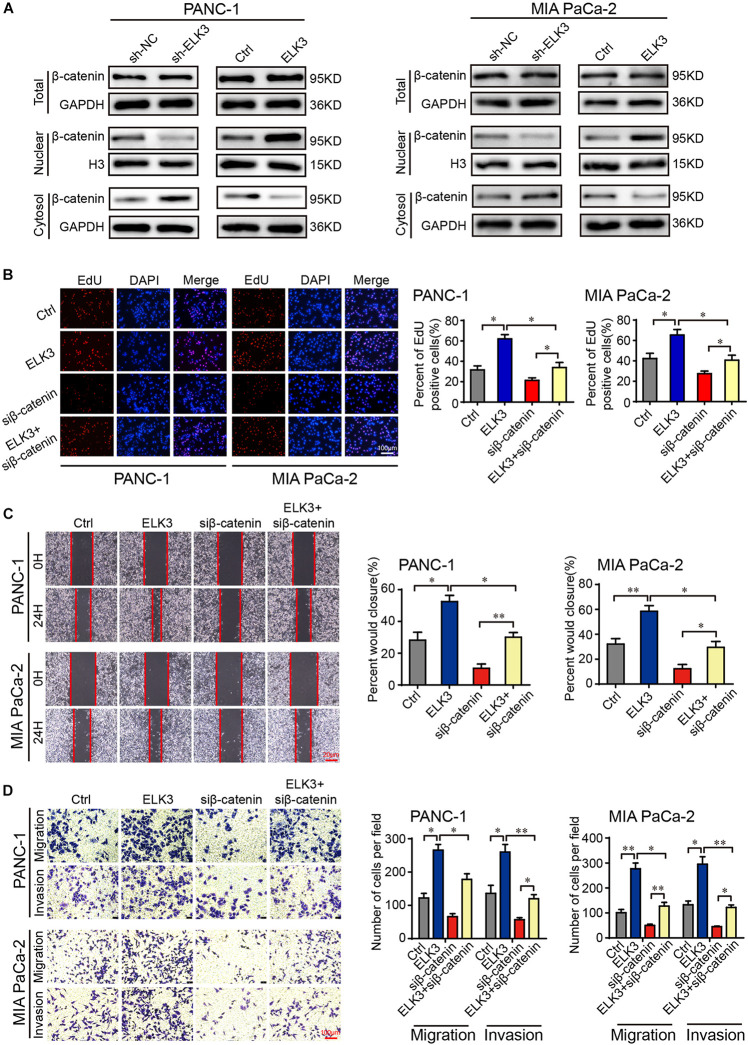
ELK3 activates the Wnt/β-catenin signaling pathway in PDAC. **(A)** The effects of ELK3 on total β-catenin, nuclear β-catenin and cytosolic β-catenin levels, as detected by western blot. **(B)** EdU assay confirming the proliferative ability of PANC-1 and MIA PaCa-2 cells treated with or without siβ-catenin (scale bar: 100 μm). **(C)** Representative images of wound healing in ELK3/PANC-1 and ELK3/MIA PaCa-2 cells treated with or without siβ-catenin (scale bar: 20 μm). **(D)** Representative images of migration and invasion in ELK3/PANC-1 and ELK3/MIA PaCa-2 cells treated with or without siβ-catenin (scale bar: 100 μm). Biological triplicate experiments were performed for each group. All data are presented as the mean ± SD. ^∗^*P* < 0.05, ^∗∗^*P* < 0.01.

Considering the importance of β-catenin signaling in the development of cancer and EMT process (Ghahhari and Babashah, 2015; Krishnamurthy and Kurzrock, 2018), we were inspired to explore whether ELK3 could regulate the Wnt/β-catenin signaling pathway in pancreatic cancer. To verify the effects of ELK3 on Wnt/β-catenin, we performed western blot assay. The results showed that neither knockdown nor overexpression of ELK3 significantly affected the total β-catenin level (Figure 5A). However, ELK3 depletion decreased the level of nuclear β-catenin and increased the cytosolic β-catenin levels, whereas ELK3 overexpression had the opposite effects on the subcellular location of β-catenin (Figure 5A). TOP-Flash and FOP-Flash luciferase reporters were used to further test the activity of the Wnt/β-catenin signaling pathway. As shown in Supplementary Figure 2, the TOP/FOP luciferase activities in PDAC cells transfected with sh-ELK3 groups were much lower than those in the control group, and were significantly higher in ELK3 overexpressed cells. Furthermore, to determine whether β-catenin is essential for the functions of ELK3, ELK3/PANC-1, and ELK3/MIA PaCa-2 cells were transfected with siβ-catenin. We found that β-catenin suppression dampened ELK3-mediated cell proliferation (Figure 5B and Supplementary Figure 3), wound healing (Figure 5C), migration and invasion (Figure 5D). These data confirmed Wnt/β-catenin signaling pathway plays a vital role in ELK3-mediated pancreatic cancer progression.

### ZEB1 Transcriptionally Activates ELK3 Expression

**FIGURE 6:**
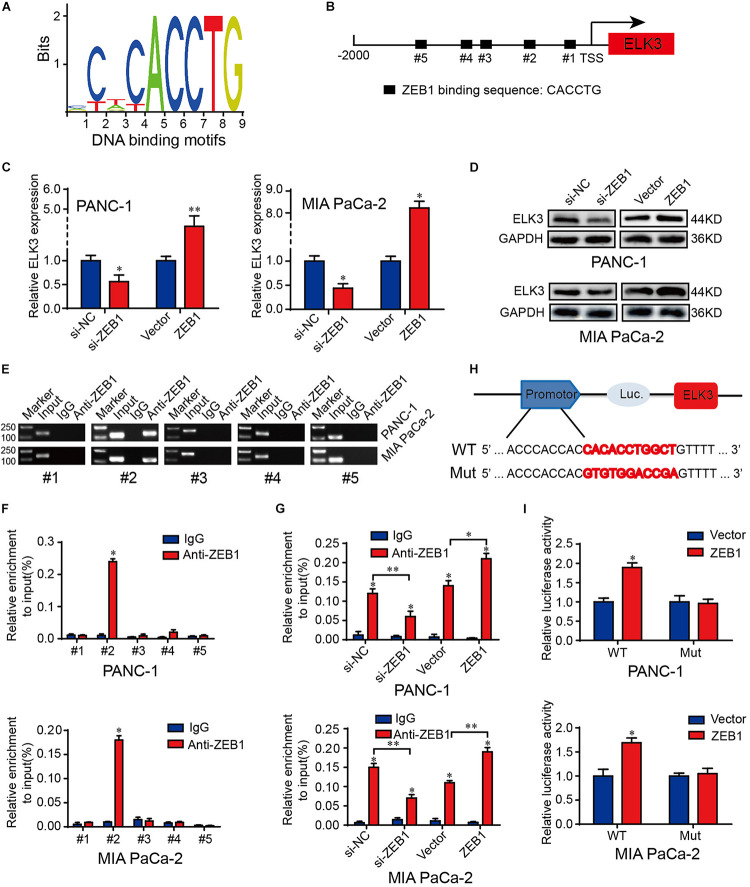
ZEB1 activates the transcriptional activity of ELK3 in pancreatic cancer. **(A)** The JASPAR database revealed the DNA-binding motifs of ZEB1 on the promoter of ELK3. **(B)** The position and binding sequences of five putative ZEB1 binding sites on the ELK3 promoter. **(C)** QRT-PCR analysis of ELK3 mRNA levels in PANC-1 and MIA PaCa-2 cells transfected with si-ZEB1 or ZEB1 overexpression plasmid. **(D)** Western blot analysis of ELK3 protein levels in PANC-1 and MIA PaCa-2 cells transfected with si-ZEB1 or ZEB1 overexpression plasmid, GAPDH was used as the loading control. **(E,F)** ChIP-qPCR experiments on five different ELK3 promoter primer using anti-ZEB1 antibody in PANC-1 and MIA PaCa-2 cells. **(G)** ChIP-qPCR experiments on the ELK3 promoter using anti-ZEB1 antibody in PANC-1 and MIA PaCa-2 cells transfected with si-ZEB1 or ZEB1 overexpression plasmid. **(H)** Schematic diagram of wild and mutant reporter plasmids. **(I)** Relative luciferase activities in PANC-1 and MIA PaCa-2 cells transfected with ZEB1 overexpression plasmid. Biological triplicate experiments were performed for each group. All data are presented as the mean ± SD. ^∗^*P* < 0.05, ^∗∗^*P* < 0.01.

To dissect the molecular mechanism of ELK3 overexpression in PDAC, we first explored the genetic or epigenetic dysregulation of ELK3 in pancreatic adenocarcinoma (TCGA, Firehose Legacy) from the cBioPortal database. However, we discovered no evidence regarding the dysregulation of ELK3 at the genetic or methylation levels (Supplementary Figure 4A), suggesting that genetic alterations (mutation, amplification, and deletion) and methylation modification may not be the main causes of the overexpression of ELK3 in PDAC. Then, we would explore its overexpression at the transcriptional level. As one of the most important EMT-inducing transcription factors, ZEB1 not only transcriptionally represses but also activates some EMT-related genes, and its overexpression promotes tumorigenesis and metastasis in human carcinomas (Wellner et al., 2009; Sanchez-Tillo et al., 2011; Krebs et al., 2017). A recent study showed that ZEB1 could collaborate with ELK3 to regulate gene expression (Cho et al., 2019). Thus, we explore whether ZEB1 could transcriptionally activate ELK3 expression in PDAC. Analyzing from the JASPAR database, we found the binding motifs of ZEB1 and five potential ZEB1 binding sites on the ELK3 promoter (Figures 6A,B and Supplementary Figure 4B). QRT-PCR and western blotting analysis demonstrated that forced expression of ZEB1 significantly increased ELK3 mRNA and protein levels, while ZEB1 deletion exhibited an opposite effect (Figures 6C,D and Supplementary Figures 4C,D). ChIP-qPCR results indicated that ZEB1 could interact with ELK3 promoter within the −641 to −631 bp region (Figures 6E,F). In addition, we found a significantly decreased ZEB1 enrichment in the ELK3 promoter following ZEB1 silencing, while ZEB1 overexpression increased the occupancy of ZEB1 in the ELK3 promoter (Figure 6G). To further investigate the regulatory role of ZEB1 on ELK3 transcription, we constructed wild-type (WT) and mutant (Mut) reporter plasmids. For the mutant plasmid, several bases were replaced in the binding site#2, and the wild type reporter contained intact binding site#2 (Figure 6H). Luciferase reporter assays showed that overexpression of ZEB1 could activate the luciferase activity of WT plasmids, but failed to activate Mut reporters (Figure 6I). To sum up, we concluded that ZEB1 binds to the region between −641 and −631 bp of the ELK3 promoter to activate its’ transcriptional activity in pancreatic cancer.

### ELK3 Is Critical for the Function of ZEB1 on PDAC Cell Proliferation and Migration

**FIGURE 7:**
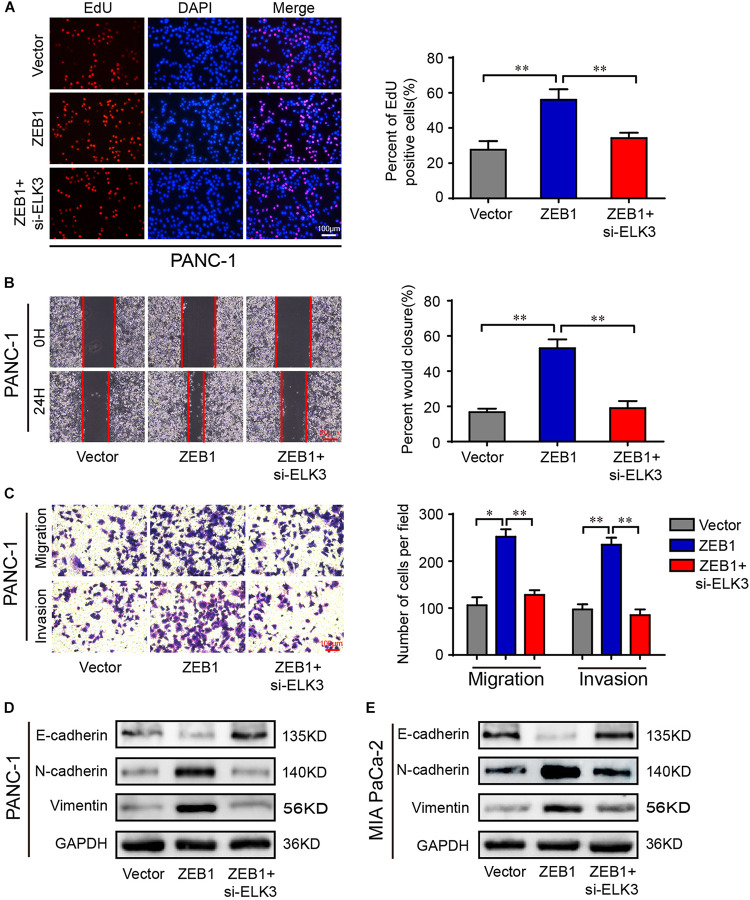
ELK3 is critical for the function of ZEB1 on PDAC cell proliferation and migration. **(A)** EdU assay analyzing the proliferative ability of PANC-1 cells (scale bar: 100 μm). **(B)** Representative images of wound healing assays performed with the indicated PANC-1 cells (scale bar: 20 μm). **(C)** Representative images of transwell migration and matrigel invasion assays performed with the indicated PANC-1 cells (scale bar: 100 μm). **(D)** Western blot analysis of E-cadherin, N-cadherin and Vimentin expression in PANC-1 cells. **(E)** Western blot analysis of E-cadherin, N-cadherin and Vimentin expression in MIA PaCa-2 cells. Biological triplicate experiments were performed for each group. All data are presented as the mean ± SD. ^∗^*P* < 0.05, ^∗∗^*P* < 0.01.

ZEB1 has been shown to promote the proliferation and metastasis of PDAC cells (Krebs et al., 2017). Since ZEB1 could increase ELK3 levels in PDAC, we investigated whether ELK3 was necessary for mediating the effect of ZEB1 on the cellular proliferation and metastasis of PDAC. As shown in Figure 7A and Supplementary Figure 5A, ZEB1-enhanced cell proliferation was inhibited by ELK3 knockdown. Moreover, ZEB1-enhanced cell migration and invasion ability was decreased when ELK3 was knockdown (Figures 7B,C and Supplementary Figures 5B,C). As an EMT-activator, western blot and immunofluorescence results showed that ZEB1 could promote the EMT process of PDAC cells, while this effect was reversed by ELK3 knockdown (Figures 7D,E and Supplementary Figures 5D,E). In summary, these results demonstrated that ELK3 was important for the oncogenic effect of ZEB1 on PDAC progression.

### Clinical Pathological Features of ZEB1 and ELK3 in PDAC Patients

**FIGURE 8:**
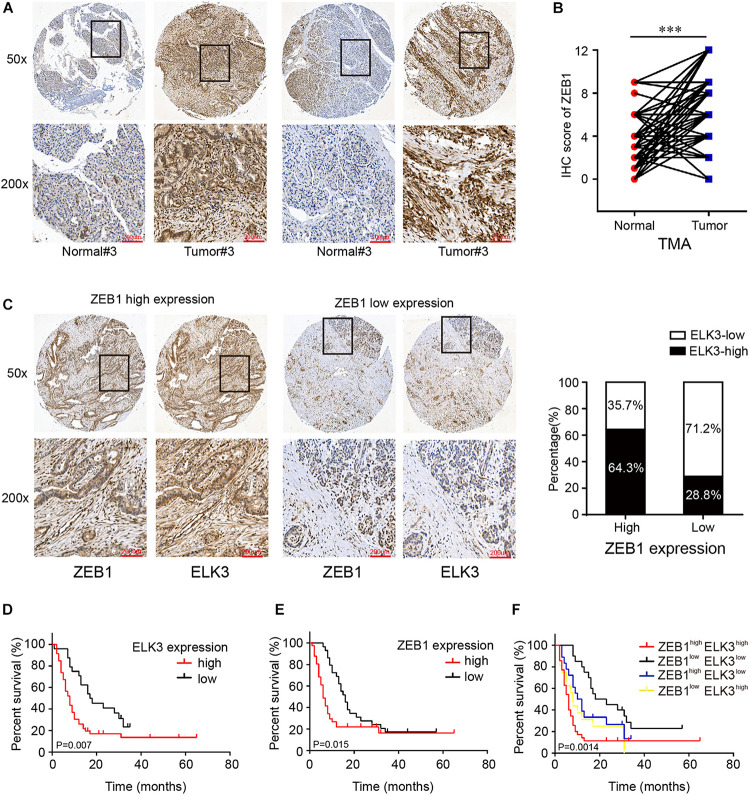
ELK3 and ZEB1 correlate closely with the clinical pathological features of pancreatic cancer. **(A)** Representative IHC images of ZEB1 on the TMA constructed from 70 pancreatic cancer tissues and adjacent normal tissues (scale bar: 200 μm; magnification: top 50× and bottom 200×). **(B)** IHC scores of ZEB1 in 70 cases of PDAC tissues with corresponding normal tissues. **(C)** Representative IHC images of ZEB1 and ELK3 in ZEB1 high expression or ZEB1 low expression tissues [scale bar: 200 μm; magnification: 50× (top) and 200× (bottom)]. **(D)** Kaplan-Meier survival analysis of the correlation of ELK3 expression level with OS of PDAC patients. **(E)** Kaplan-Meier survival analysis of the correlation of ZEB1 expression level with OS of PDAC patients. **(F)** OS analysis based on the co-expression of ELK3 and ZEB1 in 70 PDAC patients.

First, we found that ZEB1 expression was also upregulated in GSE15471 (*P* = 1.75E-07) and GSE71987 (*P* = 6.79E-06) datasets (Supplementary Figure 5A). Scatter plot analysis showed a positive correlation between the mRNA levels of ZEB1 and ELK3 (GSE15471, *R* = 0.6792, *P* < 0.0001; GSE71987, *R* = 0.8890, *P* < 0.0001) (Supplementary Figure 5B). In addition, the protein level of ZEB1 was examined in above 70 paired pancreatic cancer tissues and paracancerous tissues, and the results of IHC analysis showed that ZEB1 was highly expressed in pancreatic cancer tissues compared with matched normal tissues (Figures 8A,B). Moreover, nearly 64.3% of pancreatic cancer samples with higher expression of ZEB1 presented stronger ELK3 staining, while approximately 71.2% of those with lower ZEB1 expression exhibited weaker ELK3 staining (Figure 8C). Pearson correlation analysis confirmed the positive correlation between ZEB1 and ELK3 proteins in TMAs (*R* = 0.848, *P* < 0.0001) (Supplementary Figure 5C). Based on the median expression of ELK3 or ZEB1 in TMAs, the samples were divided into ELK3 high expression group and ELK3 low expression group or ZEB1 high expression group and ZEB1 low expression group. As shown in Table 1 and Supplementary Figure 5D, ELK3 expression was significantly higher in pancreatic cancer tissues of T3 stage, N1stage, distant metastasis M1 stage and AJCC stage IIB-IV than in these of T1–T2 stage, N0 stage, M1 stage and AJCC-IIA stage, respectively (*P* < 0.05 for all). ZEB1 expression was positively correlated with pathological grade, N stage and AJCC stage (*P* < 0.05 for all, Table 2 and Supplementary Figure 5E). Kaplan-Meier survival analysis showed that patients with higher ELK3 and ZEB1 expression levels were both associated with worse overall survival (OS) (Figures 8D,E). Moreover, the combination of these two elements demonstrated that pancreatic cancer individuals with the expression of ZEB1^high^ELK3^high^ had an even worse OS rate than any other groups (*P* = 0.0014) (Figure 8F). Taken together, ZEB1 and ELK3 predicted poor survival in clinical samples and may be indicators of efficient prognostic factors in PDAC patients.

**TABLE 1:** Correlations between ELK3 and clinicopathologic parameters in pancreatic cancer patients.

Parameters	No.	ELK3 expression		χ^2^	*P*
	(*n* = 70)	High (*n* = 46)	Low (*n* = 24)		
**Gender**					
Male	46	30	16	0.015	0.904
Female	24	16	8		
**Age (years)**					
≤60	28	18	10	0.042	0.837
>60	42	28	14		
**Tumor location**					
Head	45	27	18	1.826	0.177
Body/tail	25	19	6		
**Tumor size (cm)**					
≤3	28	15	13	3.054	0.081
>3	42	31	11		
**Pathologic grade**					
I–II	48	28	20	3.693	0.055
III	22	18	4		
**T stage**					
T1–T2	61	37	24	5.388	**0.020**
T3	9	9	0		
**N stage**					
N0	42	23	19	5.590	**0.018**
N1	28	23	5		
**M stage**					
M0	62	38	24	4.712	**0.03**
M1	8	8	0		
**AJCC stage**					
0-IIA	41	23	18	4.06	**0.044**
IIB-IV	29	23	6		
**Perineural invasion**					
Yes	30	22	8	1.353	0.245
No	40	24	16		
**Vascular invasion**					
Yes	17	9	8	1.156	0.282
No	53	37	16		

*χ^2^ text was used to test the association between categorical variables.*

*The bold values were considered statistically significant for data.*

**TABLE 2:** Correlations between ZEB1 and clinicopathologic parameters in pancreatic cancer patients.

Parameters	No.	ZEB1 expression		χ^2^	*P*
	(*n* = 70)	High (*n* = 41)	Low (*n* = 29)		
**Gender**					
Male	46	28	18	0.292	0.540
Female	24	13	11		
**Age (years)**					
≤60	28	16	12	0.039	0.843
>60	42	25	17		
**Tumor location**					
Head	45	30	15	3.403	0.065
Body/tail	25	11	14		
**Tumor size (cm)**					
≤3	28	15	13	0.481	0.488
>3	42	26	16		
**Pathologic grade**					
I–II	48	24	24	4.624	**0.0315**
III	22	17	5		
**T stage**					
T1–T2	61	35	26	0.279	0.597
T3	9	6	3		
**N stage**					
N0	42	19	23	7.693	**0.006**
N1	28	22	6		
**M stage**					
M0	62	34	28	3.115	0.078
M1	8	7	1		
**AJCC stage**					
0-IIA	41	19	22	6.100	**0.014**
IIB-IV	29	22	7		
**Perineural invasion**					
Yes	30	19	11	0.491	0.700
No	40	22	18		
**Vascular invasion**					
Yes	17	7	10	2.800	0.094
No	53	34	19		

*χ^2^ text was used to test the association between categorical variables.*

*The bold values were considered statistically significant for data.*

## Discussion

The novel ZEB1/ELK3/β-catenin axis is the crucial finding in the present report (Supplementary Figure 6). In our study, ELK3 accelerated PDAC cell proliferation, migration, invasion and EMT process *in vitro* and promoted tumor growth and metastasis *in vivo*. Mechanistic investigations suggested that ELK3 could activate β-catenin signaling pathway thereby promoting the migration and invasion of PDAC cells. Subsequent investigations revealed that ZEB1 regulated the expression of ELK3 in PDAC. Meanwhile, both were found to be overexpressed in pancreatic cancer tissues and high ZEB1 and ELK3 expression were both closely associated with patients’ clinicopathological features and worse overall survival, indicating ZEB1 and ELK3 may be efficient diagnostic and therapeutic targets in PDAC.

ELK3 is a transcriptional repressor in the subfamily of ETS domain transcription factors and contains two special inhibitory domains, the NID and CID (Buchwalter et al., 2004), and it switches to a transcriptional activator in response to activation of the MAPK/ERK1/2 pathway (Treisman, 1994). Numerous researches have reported ELK3 played important roles in various physiological processes. In breast cancer cells, ELK3 promoted the cell migration and invasion by providing oncogenic miRNAs through exosomes (Kim et al., 2019). Under hypoxic condition, ELK3 is downregulated and participates in the regulation of HIF-1α protein stability. Meantime, ELK3 also regulates hypoxic induction of genes in response to hypoxia (Gross et al., 2008). Importantly, ELK3 is required for angiogenesis, and ELK3 mutant mice exhibit decreased rate of wound closure (Zheng et al., 2003). Given the importance of ELK3 in these processes, we first examined the expression of ELK3 in GEO and TCGA databases, and found ELK3 was overexpressed in pancreatic cancer tissues compared with adjacent normal tissues. Then IHC analysis of the TMA confirmed the overexpression of ELK3 in PDAC tumor tissues. Functionally, based on the stable ELK3 knockdown and forced expression cells, we discovered that ELK3 facilitated PDAC cell proliferation, migration and metastasis *in vitro* and *in vivo*. The aberrant activation of EMT program has been proved to play an important role in the mechanisms of cancer cell migration and invasion (Thiery et al., 2009). In our present study, we found that ELK3 depletion not only significantly attenuated TGFβ-induced molecular events, but also inhibited TGFβ-induced cell wound healing, migration and invasion abilities. These data suggest that ELK3 acts as a tumor oncogene to promote pancreatic cancer progression, indicating ELK3 has the potential to be a therapeutic target for pancreatic cancer.

Wnt/β-catenin pathway, known as the canonical Wnt pathway, is overactivated in multiple cancer types, which is considered a crucial signaling pathway to accelerate the EMT process (Dongre and Weinberg, 2019). Under normal circumstances, E-cadherin and β-catenin can form a complex located at cell-cell adherent junctions in the membrane. TGFβ or EGF stimulation may activate EMT though the disassociation of this complex to release β-catenin, which then translocates into the nucleus to regulate gene expression (Xu et al., 2009). Thus, β-catenin act as a vital regulator in EMT. Our study showed that ELK3 knockdown decreased the level of nuclear β-catenin, whereas ELK3 overexpression increased the accumulation of β-catenin in the nucleus. These results suggest that ELK3 may promote pancreatic cancer progression through activating the Wnt/β-catenin pathway. To further verify this hypothesis, a series of rescue experiments were conducted. ELK3 overexpressed PDAC cells were transfected with siβ-catenin, and the results proved that β-catenin suppression could reverse ELK3-mediated cell mobility. Collectively, our data demonstrated the effects of ELK3 on β-catenin signaling, indicating the important roles of ELK3 in pancreatic cancer progression and EMT process. Emerging evidence has demonstrated the crucial rule of Wnt/β-catenin pathway in the EMT process. The study showed that the tumor suppressor gene adenomatous polyposis coli (APC) promoted the accumulation of β-catenin in the nucleus, where it interacted with T cell factor (TCF) to activate the transcription of target genes. However, whether ELK3 participates in these progresses and the specific mechanism of how ELK3 regulates β-catenin pathway need further exploration.

ZEB1, as the core EMT inducer, is a pivotal factor in tumorigenesis, invasion and metastasis in pancreatic cancer (Krebs et al., 2017). ZEB1 can regulate the expression of its target genes by recruiting corepressors or coactivators. For example, ZEB1 formed complex with NuRD contributing to the degradation of E-cadherin, thus promoting metastasis in lung cancer (Manshouri et al., 2019). In addition, ZEB1 recruited deacetylase HDAC1 and HDAC2 to attach to the E-cadherin promoter, resulting in histone deacetylation and reduction of E-cadherin expression in PDAC (Aghdassi et al., 2012). Our study verified the importance of ELK3 in the EMT process, which makes it feasible to speculate whether there is a link between ELK3 and ZEB1 during this process. Here, ZEB1 was proved to be highly expressed in PDAC and positively correlated with ELK3. Further molecular experiments demonstrated that ZEB1 could bind to ELK3 promoter and transcriptionally activate ELK3 expression. However, it is necessary to further explore whether ZEB1 can combine with ELK3, the specific mechanism of their combination and how to regulate gene expression after their combination. Considering the importance of ZEB1 and ELK3 in the EMT process and metastasis, we finally analyzed the correlation of ZEB1 and ELK3 expression with clinical pathological characteristics and prognosis of PDAC patients. We found that both high ZEB1 and ELK3 expression indicated poor prognosis.

## Conclusion

In summary, our study illustrated the oncogenic role of ELK3 in pancreatic cancer cell proliferation, migration and invasion. Overexpression of ELK3 promoted the EMT process and activated the β-catenin signaling pathway. In addition, ZEB1 upregulation contributed to the abnormal expression of ELK3. Our findings enrich the role of ELK3 in PDAC, and provide potential avenues for exploring more effective biomarkers and therapeutic strategies for the treatment of PDAC.

## Data Availability Statement

The original contributions presented in the study are included in the article/Supplementary Material, further inquiries can be directed to the corresponding author/s.

## Ethics Statement

The animal study was reviewed and approved by the Ethics Committee of Shanghai General Hospital. Written informed consent was obtained from the owners for the participation of their animals in this study.

## Author Contributions

QZ designed the experiments, performed the experiments, analyzed the data, prepared the figures, and wrote the manuscript. YR analyzed the data and designed the experiments. HX performed the experiments and proofread the manuscript. LY and WX performed the experiments and collected the clinical specimens. JL and WJ performed the experiments. ZZ proofread the manuscript. RW designed the experiments and wrote the manuscript. BL designed the experiments, wrote the manuscript, prepared the figures, and supervised the research. All authors contributed to the manuscript and approved the submitted version.

## Conflict of Interest

The authors declare that the research was conducted in the absence of any commercial or financial relationships that could be construed as a potential conflict of interest.

## Publisher’s Note

All claims expressed in this article are solely those of the authors and do not necessarily represent those of their affiliated organizations, or those of the publisher, the editors and the reviewers. Any product that may be evaluated in this article, or claim that may be made by its manufacturer, is not guaranteed or endorsed by the publisher.
