# Supplementary material for: ELK3 Mediated by ZEB1 Facilitates the Growth and Metastasis of Pancreatic Carcinoma by Activating the Wnt/β-Catenin Pathway
Source: Front Cell Dev Biol. 2021 Aug 2;9:700192. doi: 10.3389/fcell.2021.700192 (PMC8365240; doi:10.3389/fcell.2021.700192)
Supplement: Supplementary file 1 [file Data_Sheet_1.PDF]

Supplementary Figure

FIGURE S1

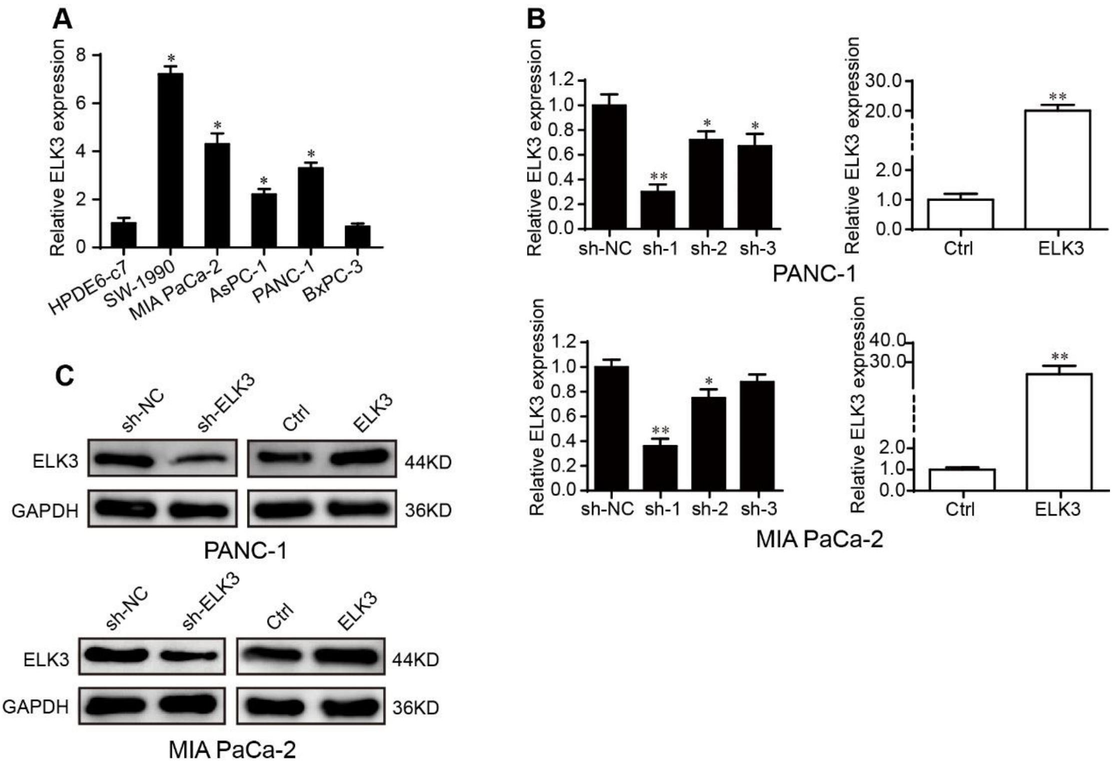

FIGURE S1 | Verify the transfection efficiency of ELK3 in PDAC cells. **(A)** QRT-PCR analysis of relative ELK3 expression in pancreatic cancer cells. **(B)** QRT-PCR analysis of relative ELK3 mRNA expression in stable PANC-1 and MIA PaCa-2 cells with ELK3 knockdown or ELK3 overexpression. **(C)** Western blot analysis confirming the ELK3 protein level in stable PANC-1 and MIA PaCa-2 cells with ELK3 knockdown or ELK3 overexpression. GAPDH was used as the loading control. Biological triplicate experiments were performed for each group. All data are presented as the mean ± SD. *P < 0.05, **P < 0.01.

FIGURE S2

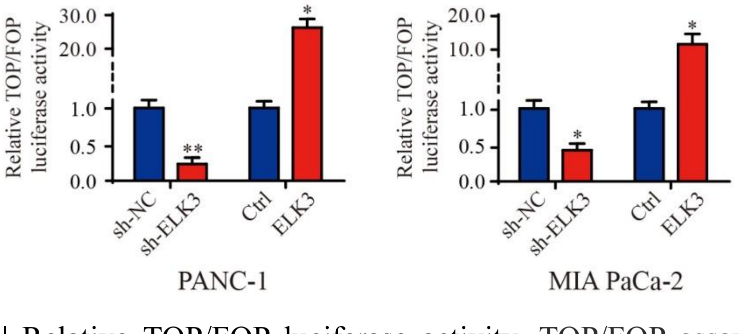

FIGURE S2 | Relative TOP/FOP luciferase activity. TOP/FOP assay showing the

activity of β-catenin in PANC-1 and MIA PaCa-2 cells after ELK3 was knockdown or overexpressed. Biological triplicate experiments were performed for each group. All data are presented as the mean ± SD. *P < 0.05, **P < 0.01.

FIGURE S3

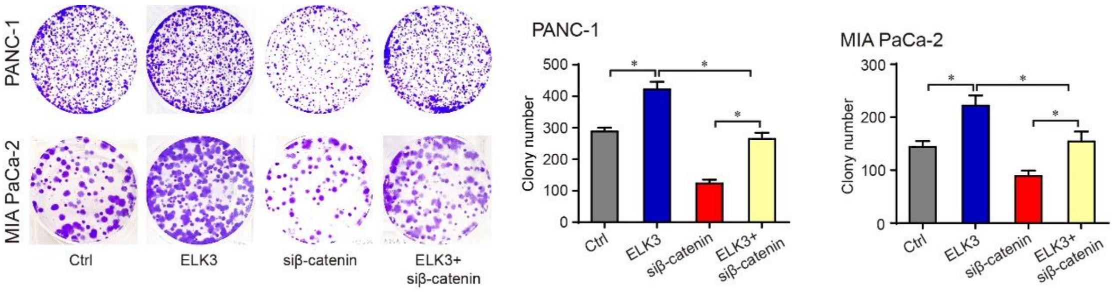

FIGURE S3 | Colony formation assay confirming the proliferative ability of PANC-1 and MIA PaCa-2 cells treated with or without siβ-catenin

FIGURE S4

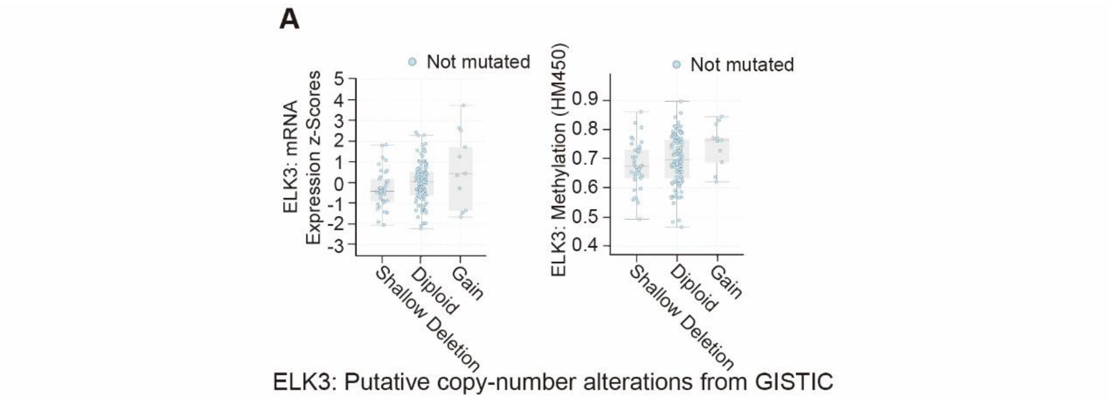

B

Matrix ID	Name	Score	Relative score	Sequence ID	Start	End	Strand	Predicted sequence
MA0103.3	ZEB1	12.1726	0.961681958	NC_000012.12:96192375-96194374	1360	1370	+	CACACCTGGCT
MA0103.3	ZEB1	11.5753	0.950663893	NC_000012.12:96192375-96194374	749	759	-	ATCACCTGGGG
MA0103.3	ZEB1	10.3855	0.928716704	NC_000012.12:96192375-96194374	482	492	+	ATCACCTGTGT
MA0103.3	ZEB1	8.97142	0.902633067	NC_000012.12:96192375-96194374	1948	1958	-	CCCACCCGCCC
MA0103.3	ZEB1	7.84841	0.881918297	NC_000012.12:96192375-96194374	677	687	+	CACACCCGGCC

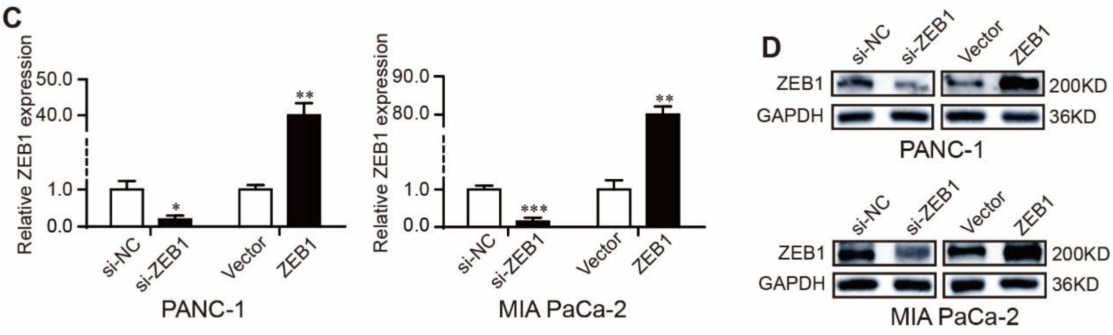

FIGURE S4 | Analyze the gene signature of ELK3 and verify the transfection efficiency of ZEB1 in PDAC cells. **(A)** The correlation of ELK3 gene expression with

its genetic and methylation levels in PDAC. **(B)** JASPAR database revealing the positions and sequences of five putative ZEB1 binding sites on the ELK3 promoter. **(C)** QRT-PCR analysis of ZEB1 mRNA in PANC-1 and MIA PaCa-2 cells transfected with si-ZEB1 or ZEB1 overexpression plasmid. **(D)** Western blot analysis of ZEB1 protein in PANC-1 and MIA PaCa-2 cells transfected with si-ZEB1 or ZEB1 overexpression plasmid, GAPDH was used as the loading control. Biological triplicate experiments were performed for each group. All data are presented as the mean ± SD. *P < 0.05, **P < 0.01.

FIGURE S5

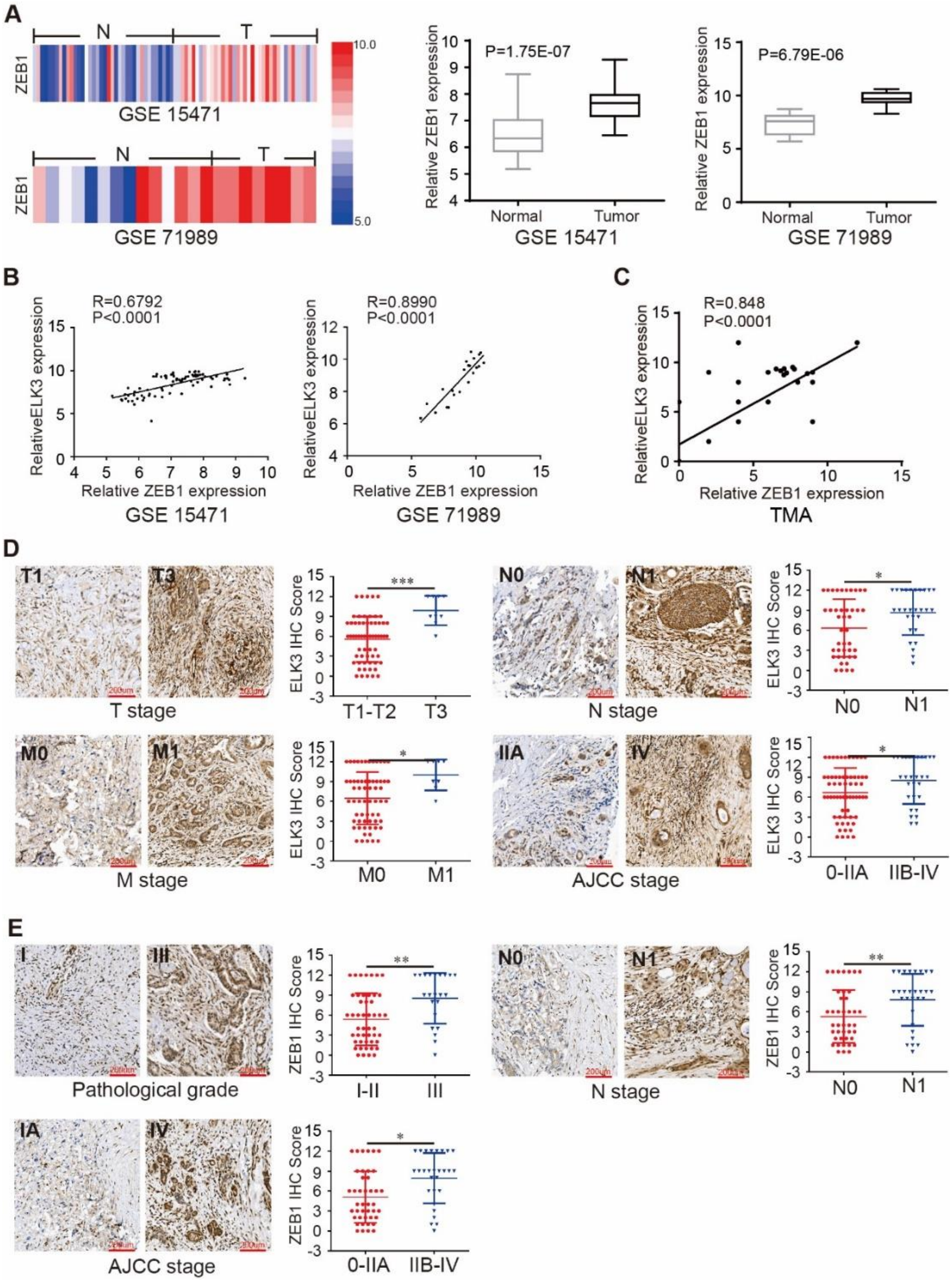

FIGURE S5 | ZEB1 expression in pancreatic cancer tissues. **(A)** ZEB1 expression level in GEO datasets GSE15471 (T, n = 36; N, n = 36) and GSE71989 (T, n = 14; N,

n = 8). **(B)** The correlation of ELK3 gene expression with ZEB1 in PDAC from GSE15471 and GSE71989. **(C)** Positive correlation between ZEB1 and ELK3 in TMAs, as detected by IHC scores (R = 0.848, P < 0.0001). **(D)** Representative IHC images and IHC scores of ELK3 in TMA samples of T1-T2 vs T3 stage, N0 vs N1 stage, distant metastasis M0 vs M1 stage and AJCC 0-IIA vs IIB-IV stage (scale bar: 200 μm; magnification: 200 x). **(E)** Representative IHC images and IHC scores of ZEB1 in TMA samples of pathological grade I-II vs III, N0 vs N1 stage and AJCC 0-IIA vs IIB-IV stage (scale bar: 200 μm; magnification: 200 x). *P <0.05, **P < 0.01, ***P < 0.001.

FIGURE S6

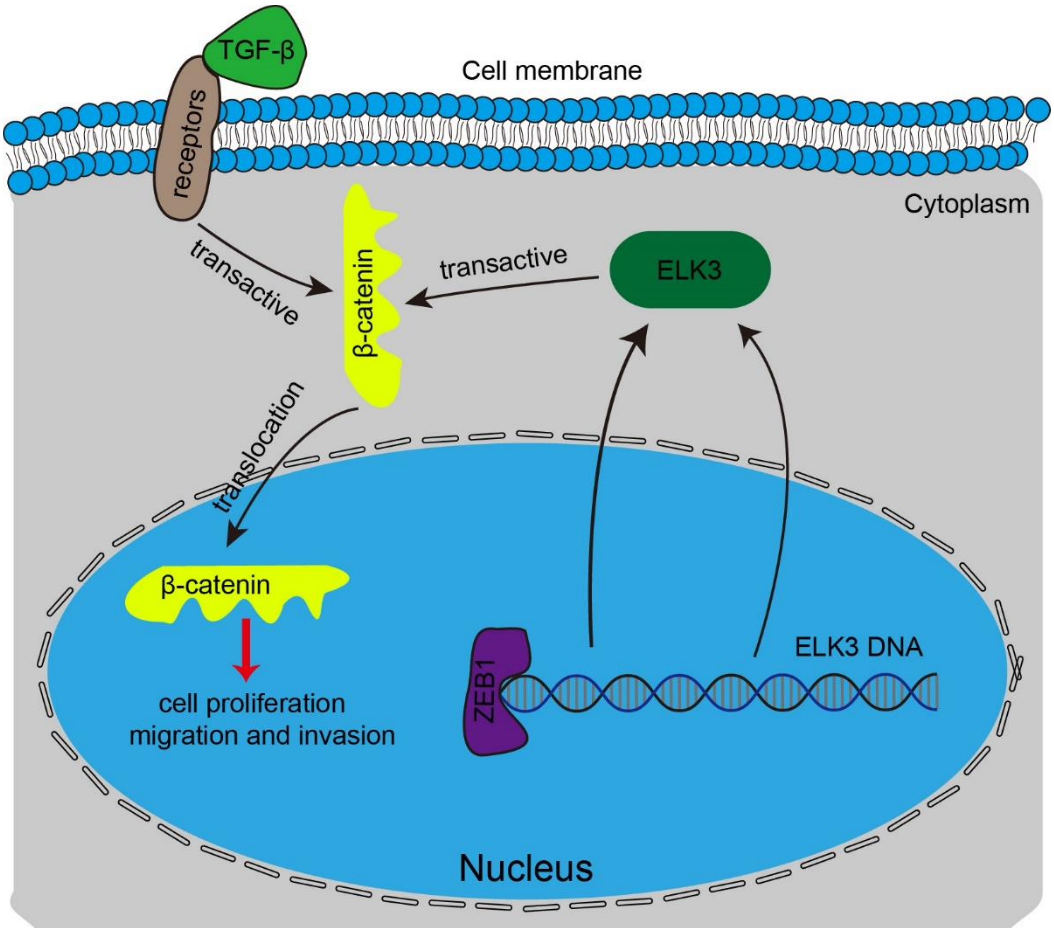

FIGURE S6 | The schematic diagram for ELK-3 induced biological function of PDAC cells.
